# Remarkable response to pericardial window procedure and weekly docetaxel treatment in a metastatic breast cancer patient with pericardial effusion and cardiac tamponade

**DOI:** 10.1002/ccr3.3380

**Published:** 2020-09-29

**Authors:** Meita Ucche, Andika Putra, Muhammad Agi Ramadhani Gustisiya, Lina Choridah, Yana Supriatna, Sri Retna Dwidanarti, Lucia Kris Dinarti, Hasanah Mumpuni, Auliya Suluk, Rusdy Ghazali Malueka, Haryo Aribowo, Suwardjo, Munawar Gani, Bambang Sigit Riyanto, Kartika Widayati Taroeno‐Hariadi, Susanna Hilda Hutajulu

**Affiliations:** ^1^ Department of Internal Medicine Siloam Hospital Yogyakarta Indonesia; ^2^ Study Program of Specialty in Internal Medicine Department of Internal Medicine Faculty of Medicine Public Health and Nursing Universitas Gadjah Mada/Dr. Sardjito General Hospital Yogyakarta Indonesia; ^3^ Study Program of Specialty in Cardiology and Vascular Medicine Faculty of Medicine Public Health and Nursing Universitas Gadjah Mada/Dr. Sardjito General Hospital Yogyakarta Indonesia; ^4^ Department of Radiology Faculty of Medicine Public Health and Nursing Universitas Gadjah Mada/Dr. Sardjito General Hospital Yogyakarta Indonesia; ^5^ Department of Cardiology and Vascular Medicine Faculty of Medicine Public Health and Nursing Universitas Gadjah Mada/Dr. Sardjito General Hospital Yogyakarta Indonesia; ^6^ Department of Anatomical Pathology Faculty of Medicine Public Health and Nursing Universitas Gadjah Mada/Dr. Sardjito General Hospital Yogyakarta Indonesia; ^7^ Department of Neurology Faculty of Medicine Public Health and Nursing Universitas Gadjah Mada/Dr. Sardjito General Hospital Yogyakarta Indonesia; ^8^ Division of Cardiac Thoracic and Vascular Surgery Department of Surgery Faculty of Medicine Public Health and Nursing Universitas Gadjah Mada/Dr. Sardjito General Hospital Yogyakarta Indonesia; ^9^ Division of Surgical Oncology Department of Surgery Faculty of Medicine Public Health and Nursing Universitas Gadjah Mada/Dr. Sardjito General Hospital Yogyakarta Indonesia; ^10^ Department of Pulmonology Dr. Sardjito General Hospital Yogyakarta Indonesia; ^11^ Division of Pulmonology Department of Internal Medicine Faculty of Medicine Public Health and Nursing Universitas Gadjah Mada/Dr. Sardjito General Hospital Yogyakarta Indonesia; ^12^ Division of Hematology and Medical Oncology Department of Internal Medicine Faculty of Medicine Public Health and Nursing Universitas Gadjah Mada/Dr. Sardjito General Hospital Yogyakarta Indonesia

**Keywords:** breast cancer, cardiac tamponade, palliative chemotherapy, pericardial effusion

## Abstract

Metastatic breast cancer may present as a pericardial effusion that can progress to a life‐threatening cardiac tamponade. Pericardial window followed by initial chemotherapy needs to be immediately applied in order to achieve a favorable outcome.

## Graphical abstract

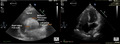

## 1 INTRODUCTION

We reported a case of metastatic breast cancer with pericardial effusion and cardiac tamponade at presentation. The life‐threatening symptoms were controlled by pericardial window procedure and weekly docetaxel chemotherapy program. Although the disease continued to progress, no signs of pericardial effusion and cardiac tamponade were identified until the patient died.

Metastatic breast cancer (MBC) at first presentation accounts for 25%‐28% of all metastatic diseases with the commonest sites being present in bones (67%), the liver (40.8%), lungs (36.9%), and the brain (12.6%).^1^, ^2^ Multiple metastases occur in about 33% of MBC patients and are associated with a 3‐year overall survival rate of 27.4%.^3^ Pericardium is not a frequent metastatic site of breast cancer and only affected in 19% of cases.^4^ About 12%‐25% of patients who have a pericardial metastasis develop pericardial effusion and only a small proportion of cases develop cardiac tamponade.^5^ Malignant pericardial effusion is considered to be an end‐stage disease because it may lead to sudden death when left uncontrolled. The prognosis of patients presenting with cardiac tamponade is gloomy, with survival ranging from a few days to 14 months, with a median survival of 5.5 months.^6^

A pericardial window and chemotherapy are essential in the treatment of cardiac tamponade. For the creation of a pericardial window, the chest is opened and a portion of the left pericardium is excised. A drain is inserted into the pleural space to drain the pericardial fluid continuously through the parietal pleura and to decrease the pressure in the pericardial cavity. The tube is left in place for a few days.^7^ Taxane‐based regimens are among the most effective and commonly used systemic therapies for breast cancer, among them are docetaxel and paclitaxel.^8^ In cases with MBC, studies observed that taxane regimens are more potent than nontaxane regimens. Docetaxel binds to tubulin, promotes the stabilization of the microtubules and causes G_2_M cell cycle arrest. Compared to paclitaxel, docetaxel showed greater affinity for the tubulin‐binding site and had more potent antitumor activity in both in vitro and in vivo models. In a phase III study, docetaxel was observed to be significantly superior to paclitaxel in terms of the improved time to progression, response duration, and overall survival.^9^ Docetaxel, as a first‐line treatment for MBC, is generally administered every 3 weeks. Compared to paclitaxel, docetaxel associates with more toxicities such as grade 3 or 4 neutropenia that occur in 93% of patients receiving it. Therefore, in specific populations such as the elderly or cases with poor performance status, weekly docetaxel is recommended, considering its comparable efficacy with lower toxicity than the standard schedule.^10^ The successful treatment of a malignant pericardial effusion in patients with breast cancer using weekly paclitaxel has been reported,^11^, ^12^ but never specifically reported using weekly docetaxel.

## 2 CASE REPORT

**FIGURE 1:**
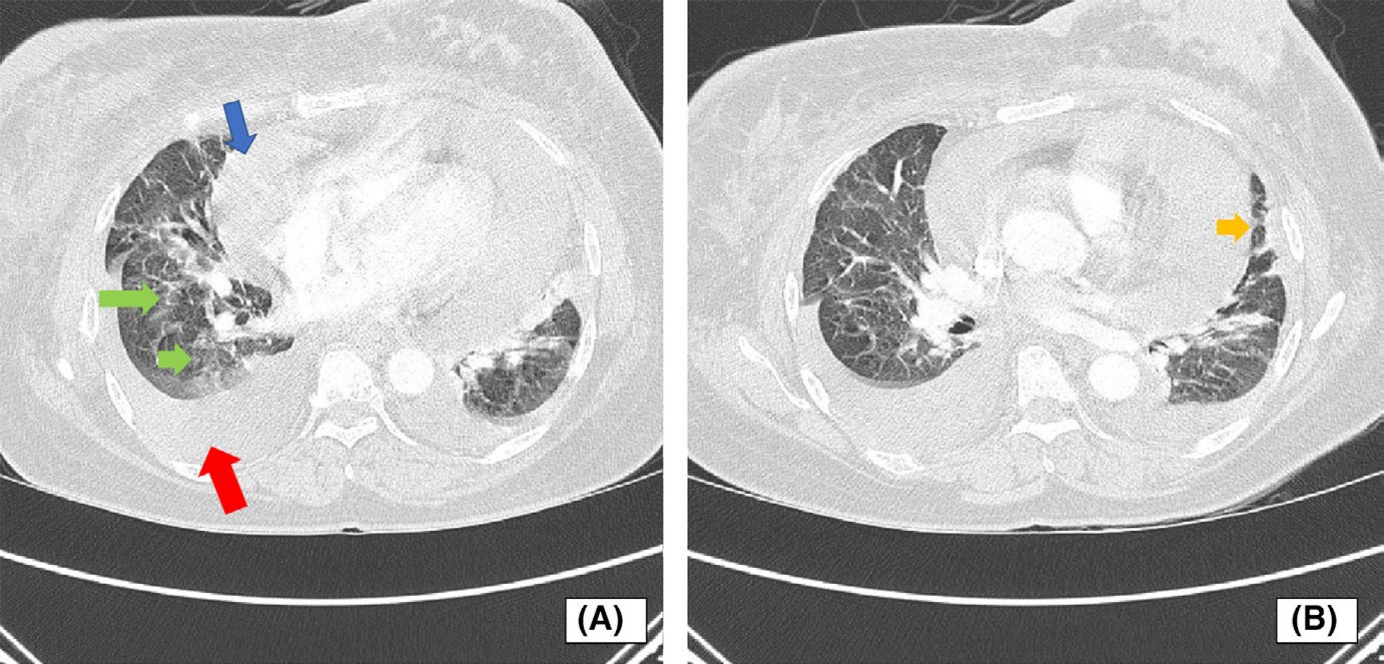
A and B, Thoracic CT scan showed pleural effusion (red arrow), pericardial effusion (blue arrow) and pneumonic type pulmonal metastases (green arrow) and subpleural type pulmonal metastases (yellow arrow). It also showed a left breast mass with irregular shape that extended to the chest wall and infiltrated to the skin and major pectoralis muscle

**FIGURE 2:**
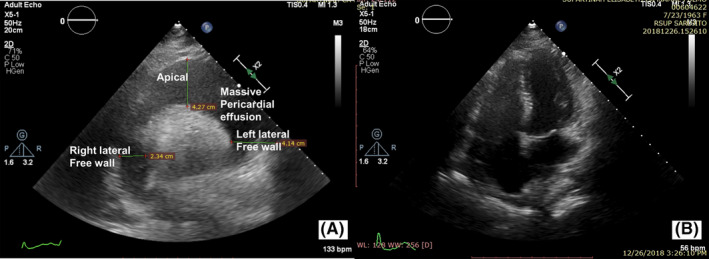
Echocardiography. A, September 2018: massive pericardial effusion during cardiac tamponade episode with ejection fraction of 50% and a decreased right ventricle function. B, December 2018: disappearance of pericardial effusion after several cycles of weekly docetaxel treatment

A 55‐year‐old woman visited our clinic in September 2018 with stage IV breast cancer. She was a mother of two sons with a history of contraceptive injection use for 5 years and suffered from type II diabetes for 4 years with routine insulin treatment. She presented with a left breast mass that had appeared several months ago. In the past 3 months, the mass grew quickly and developed a 15 × 15 cm inflammatory wound. A core needle biopsy was performed in the district hospital on June 2018 on this unresectable lesion. Pathology tests revealed ductal invasive breast cancer. An immunohistochemistry test revealed a positive estrogen receptor at 90%, a negative progesterone receptor, a negative Her2 and 75% of KI 67 expression and a poor histology grade. Her referral process to our clinic was slow because of the convoluted administration. During this process, she developed a cough. When presented in our clinic for chemotherapy she had shortness of breath. The clinical and imaging examination revealed a moderate pericardial effusion, a pleural effusion, pneumonic and subpleural type pulmonal metastases (Figure 1A,B) and bone metastasis in the form of a wedged fracture in the 12th thoracic vertebra. The pericardial effusion progressed significantly and she very quickly developed a life‐threatening cardiac tamponade (Figure 2A). She was then referred to the cardiology ward. Echocardiography revealed a massive pericardial effusion with flying heart and collapsed right atrium and right ventricle. To release the pericardial fluid, a pericardial window procedure was immediately applied. A chest tube was placed within the pericardial cavity to drain the pericardial fluid from the pericardial space. Further, cytology examination confirmed that both the pericardial effusion and the pleural effusion contained metastatic breast cancer cells. One week after the pericardial window procedure she recovered from the severe dyspnea and all the devices were then removed. A total deep vein thrombosis in her right popliteal vein was also identified and treated with unfractionated heparin followed by 20 mg daily oral rivaroxaban.

We then considered a palliative chemotherapy treatment despite her low performance (ECOG 3) because she opted for it. With consent from the patient and her family, we started a weekly intravenous docetaxel infusion^8^ with a 30 mg/m^2^ dose based on previous publication.^10^ We planned the weekly regimen for 6 cycles with 2 week rest which would be continued until disease progression. She could tolerate the first cycle of her treatment and went home as part of the ambulatory program. During subsequent treatment cycles she gradually showed an increase in her quality of life and improvement in her whole performance (ECOG 1). A remarkable response to her respiratory symptoms was noticed and after the sixth cycle of chemotherapy her chest rontgenography demonstrated the decrease of pleural and pericardial effusion. Weekly docetaxel was then continued and planned for another three cycles.

**FIGURE 3:**
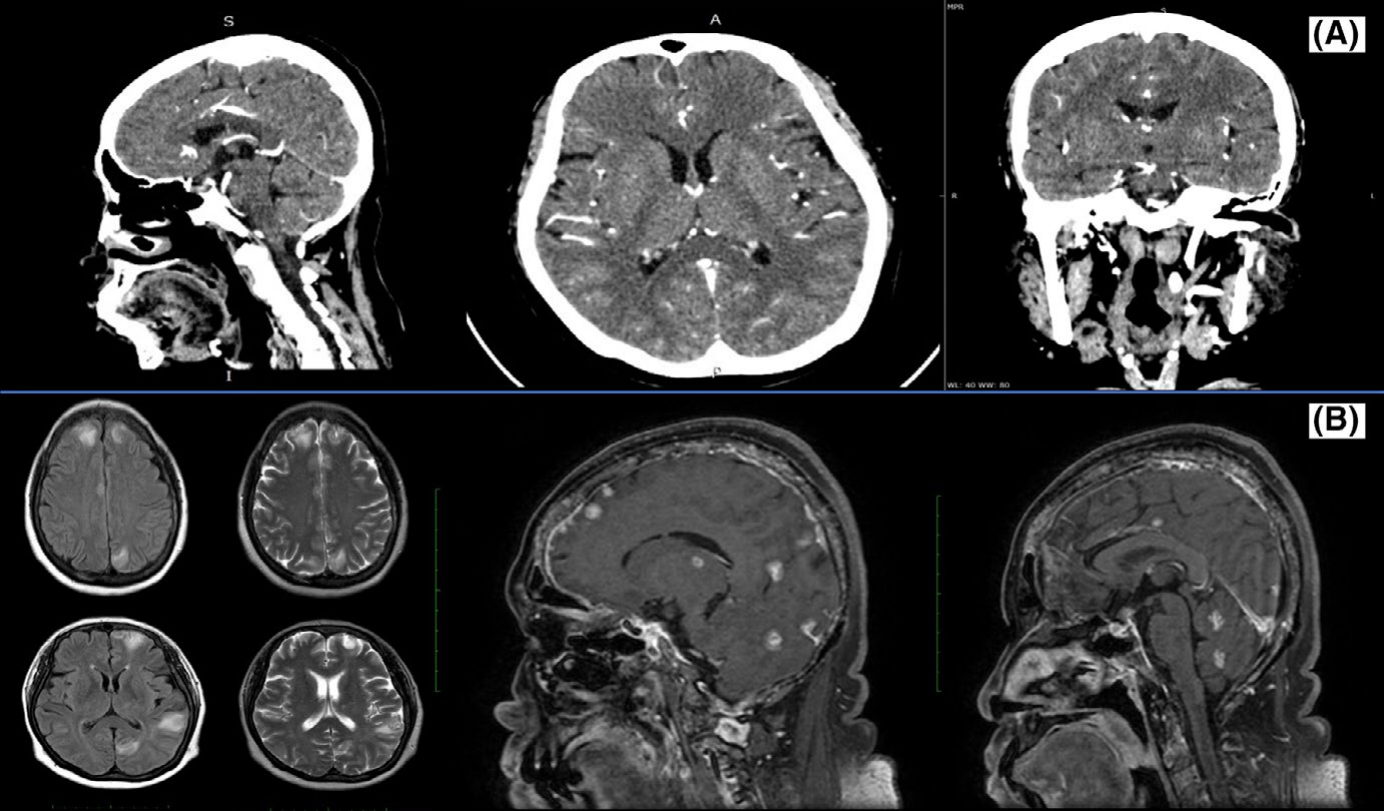
A, Head contrast‐enhanced computed tomography scan in the first seizure episode revealed cortical edema without any sign of metastasis. B, Cerebral magnetic resonance imaging in the second seizure episode showed multiple brain metastases

After the seventh docetaxel cycle, she sufferred from a cough and headache. Diagnosed as immunocompromised pneumonia she was treated with antibiotic and recovered completely. A head contrast‐enhanced computedtomography (CECT) scan was also performed to look for any signs of brain lesions. It revealed a cortical edema without any sign of brain metastasis (Figure 3A). In the ninth cycle, she experienced a generalized tonic‐clonic seizure during the administration of docetaxel without any preceeding symptom. Seizure occurred four times, for about an hour, and she fell asleep afterward. She also had a slight decrease in consciousness after the seizure. She was admitted to the hospital for brain metastasis consideration and treated with a phenitoin infusion and a dexamethason injection to relieve the neurologic symptoms. Her biochemical examination was within normal limits. An electroencephalogram showed diffuse epileptiform waves and a slowing pattern. Furthermore, her chest rontgenography showed the disappearance of the pericardial effusion that was confirmed by echocardiography (Figure 2B). The echocardiography also demonstrated dilatation of her right atrium and right ventricle with good left ventricle function and an ejection fraction of 78%. Locally, a breast ultrasound showed a decrease in the breast lump and inflammatory lesion, but a new axillary lymphadenopathy was found, confirming the progression of the disease. Doppler ultrasound of the extremities showed the disappearance of the thrombus lesion.

After full recovering from her neurologic symptoms, the patient visited our clinic with ECOG performance 2. We then planned to initiate a second‐line chemotherapy and did not give her hormonal treatment due to the consideration of brain metastases although CECT reassessment did not confirm it. Oral combination of capecitabine and cyclophosphamide is among metronomic palliative options as suggested by the guideline.^8^ Since oral cyclophosphamide was not available locally, we modified the regimen with four cycles of a 600 mg/m^2^ cyclophosphamide infusion on day 1 and a capecitabine 825 mg/m^2^ bid on day 1 to 14. The combination regimen was given at 3‐week interval. This approach was continued with metronomic capecitabine for maintenance till the disease progression. There was no dyspnea episode or signs of a pericardial effusion and seizure observed during this second‐line treatment program, and the patient's quality of life was stable. On June 2019, she started feeling frequent breast pain and had difficulty finding the words she wanted to say. She then experienced a sudden seizure episode at home, with similar characteristics to the prior event. She was readmitted for treatment of her neurology symptoms with dexamethasone and a phenytoin infusion. This time, magnetic resonance imaging (MRI) with contrast showed multi‐focal brain metastases (Figure 3B). A breast ultrasound also demonstrated additional lesions in her left breast. She received a palliative 30 Gy of whole‐brain radiotherapy (WBRT) fractionated into 10 series in July 2019. In August 2019, she started the third‐line palliative chemotherapy with paclitaxel 175 mg/m^2^ after refusing our option for a weekly navelbine regimen. Some days after receiving chemotherapy she had a sudden shortness of breath and passed away at home. The cause of death remains unclear since she was not admitted to the hospital. We assumed it might be related to the central symptom of her brain lesion.

## 3 DISCUSSION

With cardiac tamponade at presentation, the present case received initial chemotherapy to achieve rapid symptom control, as suggested by ESMO consensus guidelines for ER and/or PR positive, HER2 negative patients with visceral crisis.^8^ Taxane‐based chemotherapy is among the most effective palliative treatment for MBC. Previously, two reports have shown a good efficacy of single weekly paclitaxel therapy to relieve malignant pericardial effusions and cardiac tamponade in patients with MBC.^11^, ^12^ Ikeda et al reported it as a second‐line regimen upon relapse of the cardiac tamponade after an anthracyclin‐based treatment. Interestingly, the patient had survived for about 5 years since first diagnosis with a pericardial metastasis and received many lines of chemotherapy following paclitaxel treatment, including docetaxel. No pericardial effusion was found during the rest of the treatment,^11^ as also shown by our case. Furthermore, Einama et al observed that the time taken for paclitaxel to disappear in the pericardial effusion was shorter than for that in the plasma. This supports the notion that frequent administration during a weekly schedule effectively maintains the drug's concentration in the tissue.^12^

Docetaxel has a better antitumor activity than paclitaxel.^9^ A weekly metronomic regimen can provide favorable clinical outcome in cases with MBC, especially for unfit patients.^10^ To the best of our knowledge, there is no previous report on weekly docetaxel applications for the initial treatment of a pericardial effusion in MBC. Nevertheless, a successful outcome was demonstrated in a patient with metastatic hormone‐refractory prostate cancer with cardiac tamponade.^13^ In our present case, weekly docetaxel provided remarkable control over the pericardial effusion after the pericardial window procedure. This control persisted in the pericardium although cancer cells continued to spread locally, and to the brain. She also did not experience any neutropenia episode that frequently occur during three‐weekly docetaxel administrations,^10^ implying good tolerability for this approach.

Reasons that were considered for the general tonic‐clonic seizure during the docetaxel infusion included brain metastasis and chemotherapy side effect. Electroencephalography firstly reported diffuse epileptic waves and a slowing pattern. This was later confirmed by the MRI's description of multiple bilateral lesions after the second seizure. Some of the lesions located in deeper structures were more likely to produce their effects by irritation of the pathways conducting toward the cortex, resulting in bilateral rhythmic disturbances. The CECT scan of the brain after the docetaxel treatment only demonstrated a brain edema without metastases. However, CECT has a lower sensitivity for detecting brain metastases compared with a contrast‐enhanced brain MRI.^14^ We also performed an assessment based on the WHO‐UMC's causality assessment system.^15^ We found that the possibility of a docetaxel‐induced seizure in our patient was unlikely, considering the unclear temporal relationship between seizure and the drug administration and the occurrence of second seizure although it was withdrawn.

The present case survived 14 months after diagnosis and 11 months after cardiac tamponade presentation. When compared to the median of 5.5 months in the literature for cases with cardiac tamponade,^6^ this report demonstrated a more favorable survival. In such patient with unresectable tumor, multiple metastatic sites, an initial presentation with a malignant pleural effusion and cardial tamponade and progressivity to the central nervous system, the survival could have been worse. Beside the great effect of the palliative docetaxel treatment, the combination of cyclophosphamide and capecitabine^8^ and WBRT^16^ played an important role in her survival. As stated in the guidelines, WBRT remains the treatment of choice for patients with a poor prognosis, widely spread brain metastases, low‐performance status and uncontrolled systemic disease, with the goal being symptom control.^16^

## 4 CONCLUSIONS

The present case has shown an impressive effect of pericardial window procedure and palliative weekly docetaxel chemotherapy for controlling the symptoms of malignant pericardial effusion and cardiac tamponade. This response was durable in pericardial site despite the appearance of new metastatic lesions in the brain and reappearance of local and pulmonary lesions. Beside these approaches, subsequent chemotherapy and radiation therapy had an essential role in delivering a favorable survival in this palliative case.

## CONFLICT OF INTEREST

The authors have no conflicts of interest to declare.

## AUTHOR CONTRIBUTIONS

MU: involved in the conception of the work, the acquisition of data, and wrote the manuscript as primary author. AP: involved in the conception of the work, the acquisition of data, and wrote the manuscript. MARG: took care of the patient, involved in the acquisition of data and reviewed the manuscript. LC: acted as radiologist, have made substantial contributions to the conception of the figures and revised the manuscript. YS: acted as radiologist and analyzed and interpreted the radiology data. SRD: acted as radiation oncologist, took care of the patient and interpreted the radiology data. LKD: acted as cardiologist‐echocardiography consultant and analyzed and interpreted the echocardiography data. HM: acted as cardiologist‐echocardiography consultant, analyzed and interpreted the echocardiography data and participated in clinical advice for the patient. AS: acted as pathologist, analyzed and interpreted the pathological data. RGM: acted as neurologist, interpreted the electroencephalography data, took care of the patient, involved in the conception of the work and wrote the manuscript. HA: acted as thoracic surgeon, took care of the patient and revised the manuscript. S: acted as oncology surgeon, took care of the patient and analyzed and interpreted clinical data. MG: acted as pulmonologist, took care of the patient and analyzed and interpreted clinical data. BSR acted as pulmonologist, took care of the patient and analyzed and interpreted clinical data. KWT acted as medical oncologist, participated in clinical advice for the patient, involved in the conception of the work and revised the manuscript. SHH: acted as medical oncologist, took care of the patient, involved in the conception of the work and the acquisition of data, interpreted clinical data, drafted the manuscript and revised it for intellectual content. All authors: gave final approval of the version to be published.

## ETHICAL APPROVAL

Written informed consent was obtained from the patient's son for the publication of this case report and any accompanying images.
